# Assessment and correlates of autistic symptoms in Schizophrenia Spectrum Disorders measured with the PANSS Autism Severity Score: A systematic review

**DOI:** 10.3389/fpsyt.2022.934005

**Published:** 2022-08-30

**Authors:** Gabriele Nibbio, Stefano Barlati, Irene Calzavara-Pinton, Nicola Necchini, Elena Invernizzi, Dario Dell'Ovo, Jacopo Lisoni, Giacomo Deste, Antonio Vita

**Affiliations:** ^1^Department of Clinical and Experimental Sciences, University of Brescia, Brescia, Italy; ^2^Department of Mental Health and Addiction Services, ASST Spedali Civili of Brescia, Brescia, Italy

**Keywords:** schizophrenia, autism, PAUSS, cognition, functioning, neurobiology

## Abstract

Schizophrenia Spectrum Disorders (SSD) and Autism Spectrum Disorders (ASD) are considered separate entities, but the two spectra share important similarities, and the study of these areas of overlap represents a field of growing scientific interest. The PANSS Autism Score (PAUSS) was recently developed specifically to assess autistic symptoms in people living with SSD reliably and quickly. The aims of the present systematic review were to provide a comprehensive assessment of the use of the PAUSS scale in available literature and to systematically analyze cognitive, functional and neurobiological correlates of autistic symptoms measured with this instrument in SSD. The systematic literature search included three electronic databases (PubMed, Scopus and PsycINFO) as well as a manual search in Google Scholar and in reference lists of included papers. Screening and extraction were conducted by at least two independent reviewers. Out of 213 identified records, 22 articles referring to 15 original studies were included in the systematic review. Studies were conducted in several different countries by independent groups, showing consistent scientific interest in the use of the scale; most works focused on cognitive and functional correlates of ASD symptoms, but some also considered neurobiological features. Results of included studies showed that autistic symptoms in people with SSD are consistently associated with worse cognitive performance, especially in the social cognition domain, and with worse psychosocial functioning. However, the presence of autistic symptoms appears to also have a protective role, particularly on functioning, in subjects with more severe psychotic symptoms. Further exploring the impact of autistic symptoms could be of significant scientific and clinical interest, allowing the development of tailored interventions to improve treatment for people living with SSDs.

## Introduction

### Background

Schizophrenia Spectrum Disorders (SSD) and Autism Spectrum Disorders (ASD) are currently considered two distinct entities. According to the fifth edition of the Diagnostic and Statistical Manual of Mental Disorders (DSM-5), SSD belong to a different section with respect to ASD, which are described in the chapter detailing neurodevelopmental disorders (1). The eleventh revision of the International Classification of Diseases (ICD-11) also supports this distinction (2).

The two spectra are characterized by some remarkably different features, such as the age of onset, the course of the disorder, the response to treatment, and the fact that the presence of psychotic symptoms is not essential for a diagnosis of ASD; in particular, the age in which the first symptoms can be observed represents a very important difference in a clinical and diagnostic perspective. These distinctions are also associated with specific neurobiological and genetic characteristics, leading some researchers to theorize that SSD and ASD represent opposite models of neurobiological alteration (3).

Although these distinctions are of clinical relevance, the two spectra also share many common features: in fact, the term “autism” was developed more than a century ago by Eugen Bleuler to describe one of the four essential aspects of schizophrenia, and only in 1943 with Leo Kanner it was associated with a distinct set of clinical and behavioral characteristics that could be observed in children, leading to the subsequent conceptualization of ASD (4–7).

For instance, alterations in social interaction that can be observed in ASD are similar to schizophrenia's negative symptoms, and alterations in non-verbal communication are comparable to schizophrenia's social isolation; the absence of social and emotional reciprocity that is typical in ASD seems to match delusional patients' affective blunting, and the stereotyped language and behavior of ASD seem to recall schizophrenia's thought and behavioral disorganization (8–10).

These observations are also supported by epidemiologic evidence. In a vast cohort of patients recruited by the National Institute of Health, 30% of young subjects with early-onset schizophrenia presented with a concomitant diagnosis of ASD (11).

Moreover, several studies showed that subjects with a childhood diagnosis of autism are frequently diagnosed with a SSD during adolescence and early adulthood (10, 12–15).

Some studies also observed that the presentation of early onset schizophrenia in younger patients, especially before the onset of hallucinations and/or delusions, is difficult to clinically differentiate from ASD (16, 17).

Not only these disorders have common clinical features, but they also share important correlates in different cognitive domains: a recent study underlined that cognitive deficits measured with the MATRICS Consensus Cognitive Battery (MCCB) are present in both disorders and have similar characteristics (18). These results were also confirmed by a recent meta-analysis analyzing six different studies: while notable differences were observed in the domains of executive function and visuospatial perception (with better performances in patients with ASD), minimal differences were found for working memory and verbal skills, and similar performances were observed in processing speed and verbal comprehension domains (19).

In particular, the cognitive domain where important overlaps between SSD and ASD can be observed is the domain of social cognition: deficits in this area are a central characteristic of both disorders (20–22).

A meta-analytic work including 19 different studies comparing social-cognitive performances in subjects with SSD and subjects with ASD, showed how social cognition deficits are similar in the two disorders: no significant differences in the Theory of Mind, emotional intelligence, and social skills tests were observed. Although subjects with schizophrenia spectrum disorders showed a better performance in the field of emotions' processing, this difference was modest (23).

An even more recent work evaluated more comprehensively the social-cognitive performance in subjects with SSD, ASD and healthy controls, confirming that the level of social-cognitive impairment is very similar in the two disorders, showing minimal differences that became non-significant when corrected for symptom severity (24).

Finally, different overlaps between the two spectra can be observed at a neurobiological level: if some important differences are present when considering neural network connectivity (25, 26) and the somatosensory cortex (27), in both groups a decreased thalamic volume and functioning was observed (28–30), as well as a reduced activation of the amygdala in the act of processing social stimuli (31, 32). Finally, it appears that both disorders are characterized by a gray matter volume reduction in the temporal lobes and in the cerebellum, associated with an increase of the gray matter in the striatal areas (33).

From a genetic perspective, both disorders are often present as comorbid conditions in genetically determined neurodevelopmental disorders, such as in the 22q11.2 deletion syndrome, and in the SHANKk3 and locus 7q11.23 duplications of the Williams syndrome (34, 35).

Additionally, many alterations in known loci were found to be associated to an increased risk of developing both disorders: among these there are SNPs, copy number variations and, more rarely, chromosomal anomalies, usually related to deficits in the cyto-architectural organization of the central nervous system (36–39).

### The PANSS autism severity score

The gold standard scales for the diagnosis of an autism spectrum disorder in the general population are the Autism Diagnostic Observation Scale (ADOS) (40) and the Autism Diagnostic Interview Revised (ADI-R) (41).

The ADOS is a semi-structured observational scale, while the ADI-R is a specific structured clinical interview that is usually administered with the parents of the investigated subject. While these instruments have high validity, they are quite complex and lengthy to administer, and they are difficult to use to evaluate the autistic traits of subjects affected by schizophrenia, especially as reaching and interviewing with complex tools the parents of adults living with schizophrenia might not be considered practical in routine clinical contexts, and in some cases might not be possible at all (42). To address these issues, the Positive and Negative Syndrome Scale for Schizophrenia Autism Severity Score (PAUSS) (43) was developed.

To structure the PAUSS scale, eight specific items of the Positive and Negative Syndrome Scale (PANSS) (44) corresponding to symptoms shared in SSD and ASD were identified.

In detail, the included items are: N1 “blunted affect,” N3 “poor rapport,” N4 “passive/apathetic social withdrawal,” N5 “difficulty in abstract thinking,” N6 “lack of spontaneity and flow of conversation,” N7 “stereotyped thinking,” G5 “mannerisms and posturing,” G15 “preoccupation,” with a total score ranging from 8 to 56. The scale can also be divided in three sub-scales, based on core dimensions of ASD: “difficulties in social interactions” (N1, N3 and N4), “difficulties in communication” (N5 and N6) and “limited, repetitive and stereotypic patterns of behavior” (N7, G5 and G15).

The original validation study included a group of 1,156 patients diagnosed with schizophrenia and 256 controls diagnosed with other psychiatric disorders and with a suspected diagnosis of an ASD, 165 of which had the diagnosis confirmed during the study. The PAUSS scale showed a good convergence with the ADOS diagnosis, whereas other instruments (such as the Autism Questionnaire – AQ – and the Empathy Quotient – EQ –) didn't show similar characteristics.

Therefore, the PAUSS scale represents an accurate and practical tool and, to this day, is the only validated instrument allowing to evaluate autistic symptoms specifically in people living with SSD.

### Aims

The aims of this systematic review are to obtain a global and comprehensive evaluation of the use of the PAUSS scale to measure autistic symptoms in people living with SSD, and to comprehensively evaluate the neurocognitive, socio-cognitive, clinical, therapeutic, genetic, neuroanatomical, molecular, and neurobiological correlates of the presence of autistic symptoms, when measured with the PAUSS scale.

The main hypothesis of the present study is that the PAUSS scale, even if developed recently, has already been used in different studies from various research centers, and that there is a diverse literature allowing to define a variety of correlates of autistic symptoms.

## Materials and methods

This systematic review was conducted following the Preferred Reported Items for Systematic Review and Meta-Analyses (PRISMA) statement, using its newest edition (45, 46).

### Information sources and search strategy

A systematic review of the available literature was conducted on 3 electronic databases (PubMed, Scopus and PsycINFO) from April 19^th^, 2022, without time limitations. The following terms were used for the search: (schizophrenia OR “SSD” OR “psycho^*^”) AND (“PAUSS” OR “PANSS autism severity score”). Adaptations of the search strategy for the different databases are reported in the Supplementary Appendix 1. A supplementary search was conducted using the same terms on Google Scholar, which was also used to manually inspect all the articles citing the original validation study of the PAUSS scale and all the other included studies; reference lists of included studies were also manually inspected.

### Inclusion criteria

The inclusion criteria were defined through the PICOS Reporting System (45) as follows: regarding population (P), only studies including at least 70% of the population with a clinical diagnosis of SSD, without limitation regarding the diagnostic criteria adopted in individual studies, were considered; concerning the interventions (I) all the studies that evaluated autistic symptoms through the PAUSS scale were included; considering the original research question of this study no specific criteria for comparison (C) were selected; for the outcomes (O), all correlates of autistic symptoms that have been analyzed with the PAUSS scale, including neurocognitive and socio-cognitive performance, clinical characteristics, therapeutic, genetic, neuroanatomical, molecular, and neurobiological features were considered valid, and data regarding psychometric proprieties and validity of the PAUSS scale were also considered; finally, concerning the study design (S), controlled and non-controlled clinical trials, cohort studies, prospective case-control studies and cross-sectional studies were considered for inclusion, while reviews, case reports and case series were excluded; studies including also community or clinical controls were treated as cross-sectional studies, therefore considering the results concerning the sample of participants diagnosed with schizophrenia. Only articles published in peer-reviewed journals were selected.

### Outcomes, study selection and data extraction

Outcomes of the present systematic were the correlates of autistic symptoms in people living with SSD measured with the PAUSS: data on neurocognitive, socio-cognitive, clinical, genetic, neurobiological and treatment correlates were all taken into account, including data from the scale validation studies.

Two independent reviewers (NN and EI) assessed the reports and extracted the data; disagreements were resolved by a third author [GN. The Joanna Briggs Institute assessment for critical reviews (47, 48) was adopted to evaluate the quality of included studies, allowing to use a similar methodology even if studies with different designs emerged for the systematic literature search. Studies were considered having high methodological quality if no more than two items were had a negative rating, of acceptable methodological quality with three or four items with a negative rating, of poor methodological quality if more than four items had poor methodological quality. Scoring of each study on all items Overall quality of the evidence for the explored outcomes was assessed for consistency, precision and directness as recommended in the Grading of Recommendations Assessment, Development and Evaluation (GRADE) (49).

## Results

**Figure 1:**
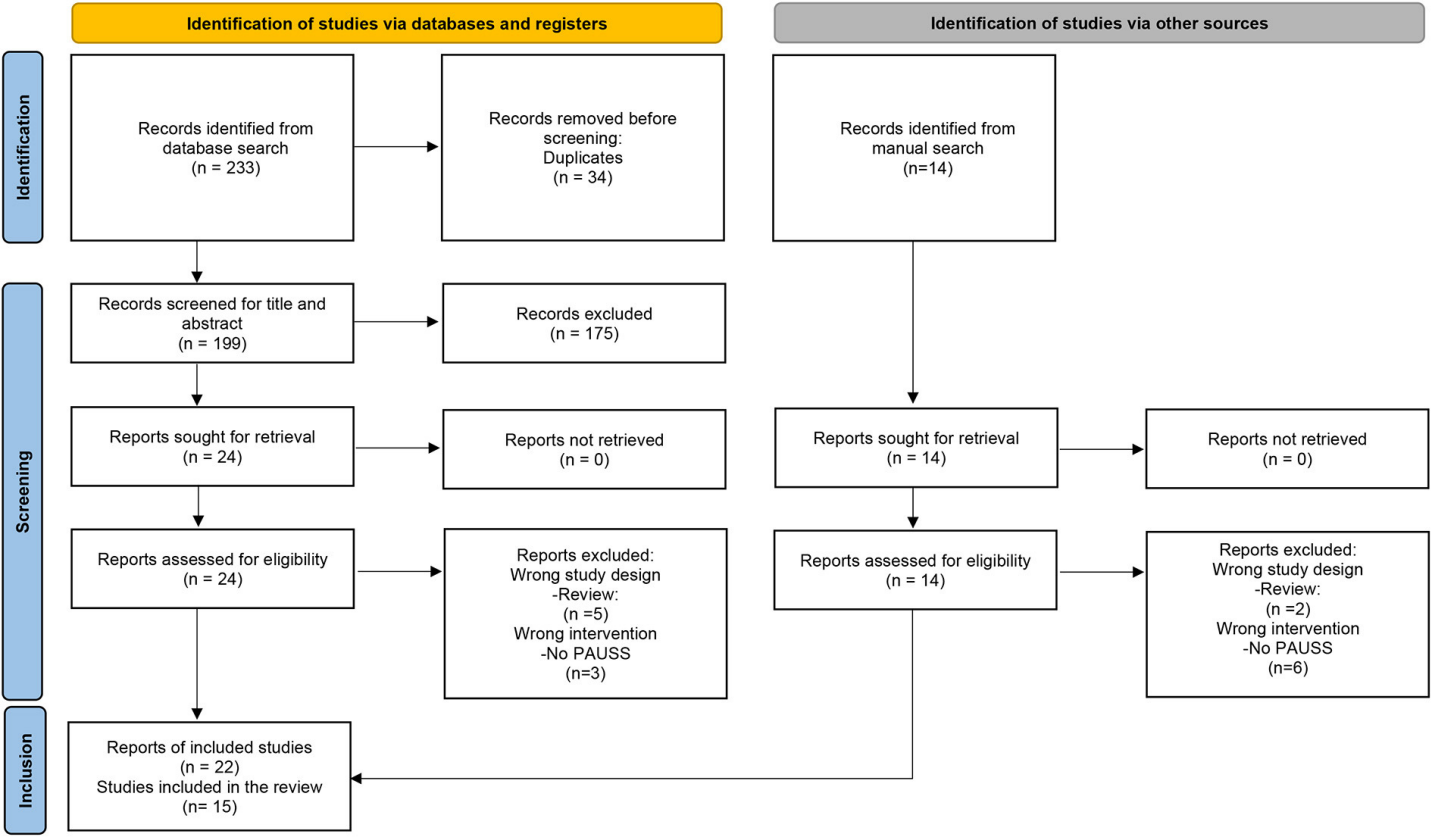
PRISMA flow diagram 2020.

The results of the systematic search are reported in Figure 1, following the PRISMA Flow Diagram 2020 indications (46).

A total of 213 original records were identified. Sixteen articles, referring to 13 primary studies, were included through electronic database search. Six additional articles, of which 4 were referred to previously included studies, were included through manual search.

At the end of the screening procedure a total of 22 papers referring to 15 studies was included in the systematic review.

### Included studies

Eight studies were conducted in Italy, one in Germany, two in Spain, one in Norway, one in Scotland, one in the United States of America and one multicentric study included participants from Germany and the United Kingdom. The studies performed in Italy were ideated and performed by four independent groups.

The patients' samples of the included projects were mostly small: all but four included <100 subjects.

The larger studies were the original validation study for the PAUSS scale (43) which included a sample of 1,156 subjects affected by schizophrenia which were carefully evaluated for the Göttingen Research Association for Schizophrenia (GRAS) study (50). The second large study was a multicentric study performed in the US which had as a primary outcome to analyze the accuracy of the scales available to evaluate social cognition in patients affected by Schizophrenia Spectrum Disorders: the Social Cognition Psychometric Evaluation (SCOPE) study (51, 52), including 361 patients from the Southern Methodists University, the Miami Miller School of Medicine and the Dallas' University of Texas. The third large study included 921 subjects diagnosed with schizophrenia and was performed by the Italian Network for Research on Psychoses (INRP): this multicentric study includes participants from 26 Italian university clinics and uses a variety of measures to evaluate cognitive performances, functional abilities and real world functioning (53, 54). The data regarding the autistic symptoms measured with the PAUSS scale were then separately presented (55). Finally, the fourth large study was a recent multicentric investigation (56) including 299 individuals diagnosed with schizophrenia and 99 individuals with first episode psychosis that were assessed with the PAUSS and different measures of functioning; this study also included 142 healthy controls drawn from a previous study (57) investigating the effects on cognition of specific copy number variants.

A description of the main characteristics of included studies is reported in Table 1.

**Table 1:** Summary of included studies.

**References**	**Country**	**Number of participants with SSD**	**Diagnosis**	**Study type**	**Other participants**	**Assessment tools**	**Outcomes (correlates of autistic symptoms)**	**Study quality**
Abu-Akel et al. (56)	Scotland	29	SPD	Cross-sectional	26 ASD, 23 healthy controls	ADOS-G, SCID-II, WAIS, SART	Cognitive correlates	High
Abu-Akel et al. (58)	Germany, United Kingdom	398	SCZ, FEP	Cross-sectional	142 healthy controls	GAF, MAS-A, SOFAS	Functional correlates	High
Barlati et al. (59)	Italy	94	SCZ	Cross-sectional	–	CGI, DAI-10, GAF, ISMI, LUNSERS, SWN-K	Clinical correlates, functional correlates	High
Bechi et al. (60)	Italy	97	SCZ	Cross-sectional	66 healthy controls	BACS, PST, PAS	Functional correlates, social cognition correlates	High
Bechi et al. (61)	Italy	96	SCZ	Non-controlled trial (cognitive remediation intervention targeting Theory of Mind)	–	BACS, WAIS-R, PST, PAS	Cognitive correlates, functional correlates, social cognition correlates, treatment response	High
Bechi et al. (62)	Italy	170	SCZ	Cross-sectional	–	WAIS-R, QLS	Functional correlates	High
Bechi et al. (63)	Italy	123	SCZ	Cross-sectional	–	WAIS-R, BACS, PST, IRI, QLS, UPSA-B	Functional correlates	High
Deste et al. (42)	Italy	75	SCZ	Cross-sectional	–	ADOS, ADI-R, WAIS, FEIT, GAF, HONOS	Functional correlates, scale validity	High
Deste et al. (64)	USA	361	SCZ, SAD	Cross-sectional	–	WRAT-3, BLERT, ER-40, EYES, HINTING, TASIT	Social cognition correlates	High
Deste et al. (65)	USA	361 (corresponds to (64))	SCZ, SAD	Cross-sectional	–	WRAT-3, UPSA-B, SSPA, BLERT, ER-40, EYES, HINTING, TASIT, SLOF	Functional correlates	High
Deste et al. (66)	USA	361 (corresponds to (64))	SCZ, SAD	Cross-sectional	–	WRAT-3, UPSA-B, SSPA, BLERT, ER-40, EYES, HINTING, TASIT, SLOF	Clinical correlates, cognitive correlates, functional correlates, social cognition correlates	High
Ehrenreich et al. (67)	Germany	1,106 [subset of Kastner et al. (43)]	SCZ	Cross-sectional	1,259 healthy controls, 2,400 subjects form the general population, 65 non-SCZ patients, 81 ASD	ACS, BDI, TICS	Genetic correlates	High
Harvey et al. (68)	USA	177 (subset of (64))	SCZ, SAD	Cross-sectional	–	BDI-2, OSCARS, SLOF	Clinical correlates, cognitive correlates, functional correlates, social cognition correlates	High
Kastner et al. (43)	Germany	1,156	SCZ	Cross-sectional	165 ASD, 100 Non-ASD, Non-SCZ	ADOS, WAIS-R, GAF, AQ, EQ	Clinical correlates, scale validity	High
Mitjans et al. (69)	Germany	1,105 [subset of Kastner et al. (43)]	SCZ	Cross-sectional	2,359 healthy controls		Genetic correlates	High
Oliveira et al. (70)	Germany	20 [subset of Kastner et al. (43)]	SCZ	Non-controlled trial (TMS)	–	GAF, MEP, EMG	Neurobiological correlates	High
Palumbo et al. (71)	Italy	77	SCZ	Cross-sectional	28 BD	ARS, BNSS	Clinical correlates, scale validity	High
Parellada et al. (72)	Spain	29	FEP	Cross-sectional	30 ASD, 26 healthy controls	WISC-R o WAISS, CGI, C-GAS, MRI scanner	Neurobiological correlates	High
Pina-Camacho et al. (73)	Spain	26	SCZ, SAD, schizophreniform disorder	Cross-sectional	33 ASD	ADOS-G, ADI-R, SRS, C-PAS, C-GAS/GAF, CGI	Functional correlates, scale validity	High
Stepniak et al. (74)	Germany	1,318 [corresponds to Kastner et al. (43)]	SCZ	Cross-sectional	111 other psychiatric diagnoses, 2,005 general population	CNI, cognitive composite score	Genetic correlates	High
Vaskinn and Abu-Akel (75)	Norway	81	SCZ, SAD	Cross-sectional	–	MASC, GAF, SFS	Cognitive correlates, social cognition correlates	High
Vita et al. (55)	Italy	921	SCZ	Cross-sectional	–	MCCB, UPSA-B, SLOF	Clinical correlates, cognitive correlates, functional correlates, social cognition correlates	High

ACS, Affective Composite Score; ADI-R, Autism Diagnostic Interview, Revised; ADOS, Autism Diagnostic Observation Schedule; ADOS-G, Autism Diagnostic Observation Schedule-Generic; AQ, Autism Quotient; ARS, Autism Rating Scale; BACS, Brief Assessment of Cognition in Schizophrenia; BDI, Beck Depression Inventory; BDI-2, Beck Depression Inventory, second edition; BLERT, Bell Lysaker Emotion Recognition Task; BNSS, Brief Negative Symptoms Scale; C-GAS, Children's Global Assessment of functioning Scale; CGI, Clinical Global Impression; CNI, Cambridge Neurological Inventory; C-PAS, Cannon-Spoor Premorbid Adjustment Scale; DAI-10, Drug Attitude Inventory short-form; EMG, Surface electromyography; EQ, Empathy Quotient; ER-40, Penn Emotion Recognition Text; EYES, Reading the Mind in the Eyes Test; FEIT, Facial Emotion Identification Test; FEP, First Episode Psychosis; GAF, Global Assessment of Functioning; GWAS, Genome Wide Association Studies; HINTING, Hinting Task; HONOS, Health Of The Nation Outcome Scale; IRI, Interpersonal Reactivity Index; ISMI, Internalized Stigma of Mental Illness; LUNSERS, Liverpool University Neuroleptic Side Effect Rating Scale; MAC-A, Metacognition Assessment Scale Abbreviated; MASC, Movie for the Assessment of Social Cognition; MCCB, MATRICS consensus cognitive battery; MEP, motor evoked potentials; MRI, Magnetic Resonance Imaging; OSCARS, Observable Social Cognition Rating Scale; PANSS, Positive And Negative Syndrome Scale; PAS, Premorbid Adjustment Scale; PAUSS, PANSS Autism Severity Score; PST, Picture Sequencing Task; QLS, Quality of Life Scale; SART, Sustained Attention Response to Task; SCID-II, Structured Clinical Interview for DSM-IV Axis II Disorders; SFS, Social Functioning Scale; SLOF, Social Level of Functioning Scale; SNP, Single Nucleotide Polymorphism; SRS, Social Responsiveness Scale; SOFAS, Social and Occupational Functioning Assessment Scale; SSPA, Social Skills Performance Assessment; SWN-K, Subjective Well-Being Under Neuroleptic Treatment Scale short form; TASIT, Awareness of Social Inferences Test; TICS, Trier Inventory for the Assessment of Chronic Stress; UPSA-B, UCSD Performance based Skills Assessment-Brief; WAIS, Wechsler Adult Intelligence Scale; WAIS-R, Wechsler Adult Intelligence Scale, Revised; WRAT-3, Wide Range Achievement Test-3.

Results of included studies are summarized in Table 2.

**Table 2:** Results of included studies.

**References**	**Results**
Abu-Akel et al. (56)	Concurrent elevated levels of autistic and positive symptoms appear to be associated to better sustained attention, but not to better attentional inhibition
Abu-Akel et al. (58)	Concurrent elevated levels of autistic and positive symptoms are associated with intermediate levels of functional impairment, while high levels of autistic or positive symptoms alone are associated with high levels of impairment
Barlati et al. (59)	Individuals with more severe autistic symptoms showed fewer years of education, greater symptoms severity, worse real-world functioning and better stigma resistance. No differences compared to other participants were observed regarding subjective well-being, global internalized stigma severity, internalized stigma and stereotype endorsement
Bechi et al. (60)	Individuals with more severe autistic symptoms show worse premorbid functioning and greater Theory of Mind impairments
Bechi et al. (61)	Individuals with more severe autistic symptoms show greater impairments in cognition, Theory of Mind and quality of life, as well as worse clinical characteristics (such as age of onset and duration of illness) Moreover, unlike other participants, they do not show improvements in Theory of Mind after a targeted intervention
Bechi et al. (62)	Autistic symptoms and positive symptoms are interactively associated to better quality of life in less severe cases They are instead independently associated with worse quality of life in more severe cases
Bechi et al. (63)	The PAUSS total score, difficulties in communication and difficulties in social interactions are correlated to worse functioning. Difficulties in communication and difficulties in social interactions predict worse functioning, while repetitive and stereotypic behavior predict better functioning
Deste et al. (42)	The PAUSS score is strongly correlated with ADOS and ADI-R. Individuals with autistic schizophrenia show worse psychosocial functioning
Deste et al. (64)	More severe autistic symptoms predict worse social cognition performance in Emotion Recognition and Theory of Mind
Deste et al. (65)	More severe autistic symptoms predict worse real-world functioning in social relationships
Deste et al. (66)	Individuals without autistic symptoms show a better clinical condition, better performance in global and social cognition, and better real-world functioning
Ehrenreich et al. (67)	Seven SNPs are correlated with more severe autistic symptoms
Harvey et al. (68)	More severe autistic symptoms are correlated with worse interpersonal functioning, worse social cognition and greater introspective bias regarding interpersonal and vocational functioning and overall real-world functioning
Kastner et al. (43)	Original validation study of the PAUSS
Mitjans et al. (69)	One SNP of the gene AMBRA1 is linked to more severe autistic symptoms in females
Oliveira et al. (70)	Individuals with more severe autistic symptoms show greater cortico-spinal excitability and greater intracortical inhibition. Excitation/inhibition balance is directly correlated to the severity of autistic traits
Palumbo et al. (71)	The total ARS score is not correlated to autistic features measured with the PAUSS
Parellada et al. (72)	Reduced posterior insular volume can be observed in young people with ASD and FEP- Higher PAUSS scores are correlated with smaller insular volume
Pina-Camacho et al. (73)	PAUSS scores are strongly correlated to ADOS and ADI-R scores. Higher total PAUSS scores represent and individual predictor of worse functioning
Stepniak et al. (74)	Eight SNP in “proautistic” genes belonging to the enlarged family of fragile X syndrome are correlated to higher PAUSS scores
Vaskinn and Abu-Akel (75)	High levels of positive symptoms and autistic symptoms appear to be associated with better global and social cognition functioning
Vita et al. (55)	Individuals with more severe autistic symptoms showed worse cognitive performance, worse functional capacity and worse real-world functioning in interpersonal relationships and participation in daily activities, but better social acceptability

ADI-R, Autism Diagnostic Interview, Revised; ADOS, Autism Diagnostic Observation Schedule; FEP, First Episode Psychosis; PANSS, Positive And Negative Syndrome Scale; PAUSS, PANSS Autism Severity Score; SNP, Single Nucleotide Polymorphism.

### PAUSS scale validity

Studies focused on the validity and on psychometric proprieties of the PAUSS had an overall high level of methodological quality.

The initial validation of the PAUSS scale (43) was tested in a large sample including 1,156 subjects with a diagnosis of schizophrenia, recruited in Germany for the GRAS study (50).

The scale showed good internal consistency, with a Cronbach's alpha of 0.857 and Spearman's correlations being statistically significant among all items (all *p* values < 0.00001, all coefficients positive, 16 correlations had a coefficient >0.4).

The accuracy of the PAUSS was compared to the ADOS scale, and they were strongly correlated (*p* = 10^−38^, *r* = 0.763). Every item on the PAUSS scale showed a correlation with the total ADOS score (*p* < 0.00001) with a coefficient higher than 0.4; one item (N3) had a correlation >0.5 and 5 items (N1, N4, N5, N7 and G15) >0.6.

A PAUSS score of 13.5 showed a 0.804 sensibility and a 0.680 specificity, whereas a score of 14.5 showed a 0.723 sensibility and a 0.711 sensitivity, setting the cut-off at 14 as the tipping-point between the two parameters. Then, based on the entire sample's scores' distribution, “extreme values” were set at 10 and 30, representing subjects without autistic traits (*n* = 168 of the original sample) and with autistic schizophrenia (*n* = 137 of the original sample), respectively.

Finally, the Receiver Operating Characteristics (ROC) of the ADOS scale had an Area under the Curve (AuC) of 0.916, whereas the PAUSS scale's AuC was 0.824.

A study by Deste et al. (42) confirmed the validity of the PAUSS scale with a more complex and accurate diagnostic approach, performed on a sample of 97 subjects with a diagnosis of schizophrenia and including both an evaluation with both the ADOS scale and the ADI-R clinical interview.

Subjects with a positive ADOS (*n* = 14) presented with significantly higher PAUSS scores compared to subjects with a negative ADOS (*p* = 0.003, *g* = 0.94). When compared to subjects with a negative ADOS score, subjects positive at the ADI-R (*n* = 9) had similar characteristics, even though the PAUSS score in this group was yet higher, leading to a greater effect size of the comparison (*t* = 3.04, *p* = 0.004, *g* = 1.10).

Another study (73) replicated these results, confirming their validity. In this study, young subjects with a diagnosis of either SSD (*n* = 26, 16–35 years old) or ASD (*n* = 33, 13–27 years old) were included. In the SSD group, the Cronbach's alpha of the PAUSS was 0.869. The PAUSS score was correlated with the ADOS-G score and the ADI-R scores, calculated through specific algorithms, and with the Social Responsiveness Scale (SRS; rho~0.500, *p* < 0.50 for all the correlations).

The results of all these studies homogeneously suggest that the PAUSS scale is a valid tool for the evaluation of subjects with a diagnosis of SSD: it is characterized by a good internal consistency and an optimal accuracy, making it as valid as more complex diagnostic tools.

An Italian study, including 51 subjects with a diagnosis of schizophrenia and 28 with a diagnosis of bipolar disorder with psychotic symptoms in the euthymic phase, compared the PAUSS scores with the Autism Rating Scale (ARS) scores. The overall scores of the two scales were not significantly correlated (*r* = 0.095, *p* > 0.2), whereas the ARS emerged as significantly correlated to the positive dimension of the PANSS scale (*r* = 0.50, *p* < 0.01). This result, as commented by the Authors, suggests that the two instruments have essential differences that are based on the nature of the analyzed autistic characteristics (71).

### Cognitive correlates

Cognitive correlates of autistic symptoms measured with the PAUSS scale have been evaluated in different studies, with particular attention dedicated to social cognitive performance. These studies had an overall high level of methodological quality.

In the original scale's validation study (43), the total PAUSS score was negatively correlated with the total IQ, measured with the Wechesler Adult Intelligence Scale (WAIS; *r* = −0.299, *p* = 0.00001). The score was negatively correlated with the ADOS score as well, but the correlation was weaker (*r* = −0.157, *p* = 0.025).

In the 2018 study by Deste et al. (42), subjects with autistic schizophrenia (PAUSS > 30), compared to other participants, all diagnosed with schizophrenia, showed a worse neurocognitive performance in various WAIS-R tests: number sequencing (*p* = 0.013, *d* = 0.61), vocabulary (*p* = 0.051, *d* = 0.46), arithmetic (*p* = 0.0002, *d* = 0.74), similarities (*p* = 0.043, *d* = 0.48), picture completion (*p* = 0.010, *d* = 0.61), symbol search (*p* < 0.001, *d* = 087) and block design (*p* = 0.050, *d* = 0.45). Also, patients with autistic schizophrenia presented with a worse socio-cognitive performance, measured with the Facial Emotion Identification Test (FEIT) (*p* = 0.051, *d* = 0.47).

According to the analyses performed on the SCOPE study database (which included 361 participants diagnosed with SSD) (64), the severity of the autistic symptoms measured with the total PAUSS score emerged in the linear regression models as an individual predictor of a worse performance for social cognition, both in the emotional processing domain, measured *via* the Emotion Recognition-40 (ER-40) test (*p* = 0.002, β = −0.150), and in the mental state attribution domain (also known as Theory of Mind), measured through the Hinting Task (HINTING; *p* < 0.001, β = −0.189) and The Awareness of Social Inferences Task (TASIT; *p* < 0.001, β = −0.185).

In the INRP study (55), which included 921 subjects, participants were divided in subjects without autistic symptoms (*n* = 56, PAUSS < 10), subjects with intermediate autistic traits (*n* = 679, PAUSS scores between 10 and 30) and subjects with autistic schizophrenia (*n* = 185, PAUSS > 30). Significant differences between the three groups were found: subjects with more prominent autistic symptoms showed worse cognitive performance in the processing speed (*p* = 0.010), attention (*p* = 0.011), verbal memory (*p* = 0.035), and social cognition (*p* = 0.001) domains and in the global cognition index (*p* = 0.010).

In a study including 97 participants with a diagnosis of schizophrenia (60), more severe autistic symptoms were observed in subjects with worse premorbid functioning (*p* < 0.0001). These patients also showed a worse cognitive performance in the processing speed (*p* < 0.0001), executive functions (*p* < 0.001) and social cognition (*p* < 0.0001) domains.

In another study by Bechi et al. (61), measuring the effects of a cognitive remediation intervention targeting social cognition abilities specifically in the Theory of Mind domain (96 participants, 6 weeks duration, 18 sessions lasting 1 hour), it was observed that patients with autistic schizophrenia (*n* = 23, PAUSS > 30), compared to other participants, all diagnosed with schizophrenia, had lower verbal IQ (*p* = 0.01), performance IQ (*p* = 0.01) and total IQ (*p* = 0.01). These patients also showed a worse cognitive performance in the domains of verbal memory (*p* = 0.02), working memory (*p* = 0.0005), processing speed (*p* = 0.03) and executive functions (*p* = 0.0005). Finally, these patients also showed a worse performance in the Picture Sequencing Task in the questionnaire score (*p* = 0.03), the sequencing score (*p* = 0.052) and the total score (*p* = 0.01).

Of particular interest are the results observed in this sample at the end of the cognitive remediation intervention: unlike other participants, subjects with autistic schizophrenia did not show a significant improvement in Theory of Mind abilities (time effect *p* = 0.89 vs. *p* < 0.0001; time x group effect *p* = 0.03, *F* = 4.57).

Results of different studies uniformly confirm that more severe autistic symptoms are correlated with a worse cognitive performance, particularly in the domain of social cognition. Preliminary evidence seems to suggest that more severe autistic symptoms can represent a marker of poorer response to cognitive remediation treatments, particularly for interventions targeting social cognition.

However, other studies suggest that the relationship between autistic symptoms and cognition could be more complex: in a study including 81 subjects with a diagnosis of schizophrenia or schizoaffective disorder (75), a combination of more severe positive symptoms (measured with the PANSS scale) and more severe autistic symptoms (measured with the PAUSS scale) symptoms were correlated to a better social cognition performance, measured through the Movie for the Assessment of Social Cognition (MASC; *p* = 0.035). Also, the group with the more severe autistic symptoms showed a reduction in the number of errors due to over-mentalization (*p* = 0.002).

Additionally, similar results were observed in a group of 29 patients affected by schizotypal personality disorder (nine of whom also had a diagnosis of ASD) (58): a high score at the positive sub-scale of the PANSS scale, together with a high score at the PAUSS scale, were associated to a smaller number of omission errors in the random version of the Sustained Attention to Response Task (SART), a test measuring the sustained attention performance (*p* = 0.003).

The results of these studies suggest that autistic traits, even if correlated to a worst cognitive performance, can also play a protective role on some cognitive abilities in subjects with more severe psychotic symptoms. Although promising, these results have to be considered preliminary in light of the small number of studies investigating this topic, the small size of recruited samples and the heterogeneity in the included diagnoses.

### Functional correlates

Functional correlates of autistic symptoms represent another area of particular interest in the studies emerging from the systematic literature search. Studies focusing on functional correlates of autistic symptoms showed a high level of methodological quality.

In a study by Deste et al. (42) published in 2018, participants' psychosocial functioning in real-world activities was evaluated through the Global Assessment of Functioning (GAF) scale and the Health of the Nations Outcome Scale (HoNOS): subjects with autistic schizophrenia showed a worse psychosocial functioning both at the GAF scale, with a large effect size (*p* < 0.01, *g* = 0.99), and at the HoNOS scale, with a moderate effect size (*p* = 0.02, *g* = 0.54).

In the SCOPE study, real-world functioning was measured with the Specific Level Of Functioning (SLOF) scale. In one of the articles illustrating the results of this study (65) more severe autistic symptoms emerged as an individual predictor of a worse psychosocial functioning in the domain of interpersonal relationships (*p* < 0.001, β = −0.189).

Another paper, reporting data on a sub-group of patients from the SCOPE study (68), shows that more severe autistic symptoms are correlated with a worse interpersonal functioning (*r* = −0.40, *p* < 0.001), but also with a stronger bias in the subjective evaluation of interpersonal functioning (*r* = 0.17, *p* < 0.05) and of work abilities (*r* = 0.18, *p* < 0.05), measured as the discrepancies observed between the self-rated and the informant-rated assessments.

In a study by Bechi et al. (62), 123 subjects with diagnosis of schizophrenia were divided into patients with good (*n* = 60) and with impaired functioning (*n* = 63). Better functioning was predicted by a lower score in the PAUSS “difficulties in social interactions” subscale (*p* = 0.030, β = −0.27) and in the PAUSS “difficulties in communications” subscale (*p* = 0.002, β = −0.47). However, having a higher score at the PAUSS' “stereotyped and repetitive interests” subscale (*p* = 0.004, β = −0.28) emerged as a significant predictor for a better functioning.

In the INRP study (55) functioning was measured with the SLOF scale: patients with autistic schizophrenia showed worse psychosocial functioning when compared to patients with intermediate or absent autistic symptoms: they showed a significantly worse functional capacity (*p* = 0.004), and worse real-world functioning in interpersonal relations (*p* < 0.001) and in participation in community activities (*p* < 0.001). However, considering the correction for the psychotic symptoms' severity, they also showed better social acceptance (*p* = 0.019). This observation could be due to the style of social interaction of individuals with autistic traits, which are more prone to social isolation and could therefore be perceived as more socially acceptable compared to other patients with severe psychotic symptoms.

Even if the literature agrees that autistic symptoms are correlated to a worse psychosocial functioning, as was observed for cognitive performances, some studies show a protective effect of autistic symptoms on patients with a more severe positive symptomatology.

In a study by Vaskinn and Abu-Akel (75) the correlation between the scores obtained at the positive subscale of the PANSS scale and those obtained at the PAUSS scale was associated to higher scores at the GAF scale (*p* = 0.005) and at the Social Functioning Scale (SFS; *p* = 0.029).

Quality of life was evaluated in two studies by Bechi et al. (60, 62). In the first study (60) the evaluation was made with the Quality of Life Scale (QLS): subjects with autistic schizophrenia had a worse overall quality of life (*p* = 0.01, *F* = 6.178) and worse self-directness (*p* = 0.0005, *F* = 12.983); however, no significant difference was observed in the subscales regarding the domains of relationships and work.

In the second study (62), results regarding the QLS were similar to those observed for cognitive performance and functional outcomes: in a sample of 170 patients, worse autistic symptoms were associated with worse quality of life (*p* < 0.001, β = −0.29). However, positive symptoms' severity and autistic symptoms' severity were interactively associated in improving the quality of life of the subgroup of patients with less severe symptomatology (*p* < 0.001, β = −0.40).

A large and recent study (56) included 299 participants with chronic schizophrenia and 99 participants with first episode psychosis. Both samples were assessed with the PANSS to evaluate global and positive symptoms severity and with the PAUSS to evaluate the severity of autistic traits; functioning was assessed in participants diagnosed with schizophrenia with the GAF and those with first episode psychosis with the Social and Occupational Functioning Assessment Scale. In both samples, better functioning was predicted by a positive interaction between positive symptoms and autistic traits (β = 1.95, *p* < 0.001 for the schizophrenia sample model and β = 1.09, *p* = 0.014 for the first episode psychosis sample model): participants with either severe positive symptoms or with high levels autistic symptoms had worse functioning than participants showing high levels of both. These findings represent the strongest available evidence supporting the hypothesis that autistic symptoms have a protective effect in limiting the negative impact of psychotic symptoms on psychosocial functioning.

Finally, another recent study (59), including data on 94 individuals diagnosed with schizophrenia and assessed for a previous study (76), investigated the impact of autistic symptoms on global functioning, subjective well-being, and internalized stigma, measured with the Internalized Stigma of Mental Illness (ISMI) scale. Patients with autistic schizophrenia (PAUSS ≥ 30), compared to other participants, showed worse global functioning both a 1-week and 1-year assessment (*p* < 0.001 for both); however, no significant difference was observed in subjective well-being. and in global internalized stigma. Considering the different factors of the ISMI, no between-group differences were observed regarding experiential stigma and stereotypes endorsement: however, participants with autistic schizophrenia showed better stigma resistance (*p* = 0.022), suggesting that autistic traits could also have a protective effect in the internalization of stigma.

### Genetic and neurobiological correlates

Genetic correlates of autistic symptoms measured with the PAUSS scale have been analyzed in three investigations which are part of the GRAS study, which also allowed the original scale validation.

The work by Stepniak et al. (74) shows how eight out of the 13 Single Nucleotide Polymorphisms (SNP) analyzed (all linked to the gene family of the fragile X syndrome) are correlated to more severe autistic symptoms and are potentially “pro-autistic” genes: the presence of a higher number of genes carrying those polymorphisms, corrected for eventual confounding factors, determines an autistic phenotype, which is more severe when there is a higher number of genes carrying those SNPs (*r* = 0.103, *p* = 0.0008).

In a paper by Mitjans et al. (69) it is underlined how in females with a diagnosis of schizophrenia the intronic SNP rs3802890-AA in the AMBRA1 gene, involved in autophagy and neurodevelopment, is associated with more severe autistic traits (*p* = 0.030). This association was not significant in males (*p* = 0.101).

Finally, in a study by Ehrenreich et al. (67) various SNPs studied with different Genome Wide Association Studies (GWAS) were analyzed, with the aim of identifying a specific phenotype, the OTTO phenotype. Seven SNPs were identified as correlated with more severe autistic symptoms: the presence of a higher number of SNPs increased the severity of the autistic symptoms (*p* = 0.038). The comparison between extreme groups (subjects with 0/1 SNP vs. subjects with 6/7 SNPs) showed an even stronger correlation (*p* = 0.003).

Concerning neuroimaging, only one study using the PAUSS scale could be found: it evaluated through magnetic resonance (1.5 Tesla) 29 participants aged less than 18 years with a first psychotic episode, 30 children diagnosed with ASD and 26 healthy volunteers. In the group of patients with a psychiatric diagnosis, worse autistic symptoms were negatively correlated to the left posterior insular volume (*r* = −0.30, *p* = 0.028); this correlation remained significant also when considering the group of patients with the first psychotic episode (72).

Finally, in the study by Oliveira et al. (70), 20 male participants in the GRAS study were selected to take part in a study of transcranial magnetic stimulation (TMS) and were split into two groups, high and low autistic traits, based on the median PAUSS score. The two groups were balanced for age, dominant hand, GAF score and antipsychotic dosage. Subjects with more severe autistic symptoms showed a higher cortico-spinal excitability (*p* = 0.031) and a higher cortical inhibition (*p* = 0.044). Additionally, the inhibition/excitation proportion, calculated for each patient, was positively correlated to the severity of the autistic symptoms (*r* = 0.511, *p* = 0.021). No significant differences were observed among the groups regarding the intracortical facilitation (*p* = 0.143).

### Correlates of absence of autistic symptoms

Only one paper, derived from the SCOPE study (66), focused on the evaluation of participants without autistic symptoms (PAUSS < 10). These patients showed better premorbid IQ (*p* = 0.034, *d* = 0.278), better neurocognitive performance (*p* < 0.001, *d* = 0.616), less severe symptoms (*p* < 0.001, *d* = 1.34), better functional capacity (*p* < 0.001, *d* = 0.729), better social functioning (*p* < 0.001, *d* = 0.731), better social cognitive performance (*p* < 0.05, *d* > 0.200 in all tests) and better real world functioning in the areas of interpersonal relationships (*p* < 0.001, *d* = 0.791), community activities (*p* = 0.001, *d* = 0.435) and working activities (*p* = 0.001, *d* = 0.442). Better functional capacity (*p* = 0.002, β = 0.040), better social capacities (*p* = 0.001, β = 1.225), less severe psychotic symptoms (*p* < 0.001, β = −0.064), better neurocognitive performance (*p* = 0.002, β = 0.065), better socio-cognitive performance (*p* = 0.014, β = 0.113) and better functioning in the interpersonal relationships' areas (*p* < 0.001, β = 0.905) also emerged as predictors of absence of autistic symptoms.

### Quality of the evidence

Scoring of each study on all items of the Joanna Briggs Institute checklist is reported in the Supplementary Appendix 2.

All included studies were conducted on well-characterized and well-described samples, with clear inclusion criteria and an appropriate diagnostic evaluation. Study outcomes were well-defined and accurately assessed in all studies, and statistical analyses were appropriate. Some of the earliest cross-sectional studies did not consider some relevant confounding factors, such as potential overlaps between autistic symptoms severity and global symptoms severity, positive symptoms severity or non-autistic symptoms severity, or did not include these elements in dedicated control analyses. These elements were however taken into account in more recent studies in several different ways.

Quality of the individual outcomes results was considered high for the evidence regarding clinical, cognitive, functional and social cognition correlates of autistic symptoms measures, as the results of included studies were showed high levels of consistency, directness and precision. In particular, differences in the results of single studies that could lead to doubts regarding consistency and precision of the findings were explained by the moderating effect of positive symptoms severity, which were replicated in several studies with coherent results.

As regards genetic, neurobiological and treatment outcomes, high-quality individual studies are present but in very limited number, so further research is needed to provide comprehensive evidence.

## Discussion

Since its original validation (43), the PAUSS scale showed an ever-growing use in the fields of psychiatric research and, to a lesser extent, neurobiological research.

In this review, 22 different articles were included, describing the results of 15 studies performed in different countries by independent groups.

Most of the studies that used the PAUSS scale to evaluate autistic symptoms in schizophrenia included small samples, but some large studies were found as well. The global quality of included studies was high, and all studies showed a low risk of bias. This observation is in line with the recent publication of these works, which were all performed in the last 10 years: the fact that more recent studies are characterized by better methodological quality is well described in meta-analytic literature (77, 78).

### Correlates of autistic symptoms

Cognition, and more specifically social-cognition, and functional outcomes emerged as the most frequently explored correlates of autistic symptoms.

Included studies showed that autistic symptoms appear to have a negative impact on cognitive performance, and this result is quite homogeneous among the different works, representing one of the main topics of larger studies (55, 66). Subjects with worse autistic symptoms show a worse performance in different social cognition domains (42, 60, 61) and severe autistic symptoms represent an individual predictor of greater social cognition impairment (65).

This represents an expected result, as social cognitive deficits are one of the essential characteristics of both spectra (20–22, 79). This finding is also in line with the results of the studies that used different instruments to evaluate autistic symptoms in people with SSDs (80).

As regards functional outcomes, the presence of autistic symptoms appears to have an important negative impact: real-world functioning is globally more compromised in subjects with more severe autistic symptoms, especially when considering social functioning (42, 55, 63, 68, 73). Moreover, more severe symptoms represent an individual predictor of worse social functioning (65). These findings are in line with results of recent studies using instruments other than the PAUSS to evaluate autistic symptoms in people with SSD (80–82).

Autistic symptoms also appear to worsen quality of life in people with SSD (62, 63).

Although all the above-mentioned issues are described in most of the included studies, some evidences suggest that, when considering a specific group of patients, autistic symptoms can also have a protective effect: in cases of severe positive symptoms, autistic traits appear to be associated with better attention (58, 75), better social acceptability (55), better functioning (56, 75) and better quality of life (62).

This effect could be a consequence of the typical relational style of subjects with prominent autistic traits: being more prone to social isolation could limit their interactions with other people, leading them to be considered more socially adequate compared to other patients with severe psychotic symptoms. Additionally, if on one hand being immersed in personal autistic experiences can lead to worse functioning, on the other hand it also lead to higher bias in the evaluation of one's own abilities (68), which could somehow have a positive effect on the perception on one's own quality of life. Finally, the different impacts of autistic symptoms could also be explained by the use of different coping mechanisms in people with prominent autistic traits compared to other subjects diagnosed with schizophrenia (81).

Regarding biological correlates of autistic symptoms measured with the PAUSS scale, available literature is still limited. The most explored area is genetic research: different studies have identified polymorphisms that could be considered “pro-autistic” (67, 69, 74). This focus on genetics is probably due to the original validation study of the PAUSS scale, which did not only evaluate phenotypic characteristics but also genetic ones (43).

Regarding the study of the neuroimaging correlates, only one study was found, and it included only very young participants with a first, early-onset psychotic episode (72): the results show that a reduction of insular volume is correlated with the presence of autistic symptoms. These results are very interesting but they represent a limited contribution to the research field, as they do not allows to understand the complex neurobiological relationship that exists between psychotic and autistic features. In fact, the two spectra appear to share some characteristic alterations, such as reduced volume and functioning of the thalamus (28–30) and a reduced activation of the amygdala in the processing of social stimuli (31, 32), but they also seem to have opposite alterations in other regions, such as the somatosensory cortex (27) and, most importantly, at the level of neural networks (25, 26).

### Future perspectives

No included study provided a longitudinal evaluation of autistic symptoms, which is necessary to analyze the stability or the trend of variation of the PAUSS score. At the present moment, it is not possible to say for sure if autistic characteristics measured with the PAUSS scale represent a trait, stable in time and associated with a specific genotype and phenotype, or rather a symptomatologic dimension that could be modifiable with treatment. The genetic studies identified through the systematic search point toward the first option, but this hypothesis remains to be confirmed.

Another topic that needs to be further investigated is the relationship between negative symptoms and autistic traits, as they present several phenomenological and clinical similarities. The overlap becomes even more evident when considering that several items from the PAUSS belong to the negative sub-scale of the PANSS. Investigating more thoroughly the areas of overlap and the differences between the two domains using different measures of negative symptoms severity such as second-generation assessment tools, which are more accurate (83), represents an important future perspective.

The relationship between autistic features and stigma, and particularly internalized stigma, also requires further study. Internalized stigma represents a feature that has an important impact on the lives of people with schizophrenia, strongly influencing their identity, their functioning and their quality of life (84–86). Only one study assessed the relationship between autistic features measured with the PAUSS and internalized stigma, showing that more severe autistic symptoms are not correlated with worse internalized stigma, and could even have a protective effect on stigma resistance (59). On the contrary, the results of two studies conducted by another research group suggest that more severe autistic symptoms could be correlated to worse internalized stigma (87, 88). However, in these studies the severity of autistic symptoms was not assessed through clinical observation or caregivers reports, but though self-rated assessment tools.

Neurobiological correlates of the autistic symptoms in schizophrenia are another area of interest and should be studied more thoroughly: since functional and structural neuroimaging studies underline many similarities but also remarkable differences between the two spectra (16, 25, 27), whether people diagnosed with schizophrenia with more severe autistic symptoms have an intermediate neurobiological phenotype, a phenotype more similar to one of the two spectra, or even an entirely different phenotype remains to be verified.

Finally, whether the presence of autistic symptoms represents a marker for the response to specific treatments remains to be more thoroughly evaluated, as only one study regarding this topic emerged from the systematic literature search. Results of the study suggest that the presence of more severe autistic symptoms is correlated to lack of response to a specific cognitive remediation therapy aiming to improve social cognition in the Theory of Mind domain (61). This result is particularly interesting considering that subjects with a worse clinical presentation usually have a better response to cognitive remediation treatments (89–92). Moreover, other studies where autistic symptoms where not evaluated with the PAUSS scale show that the presence of autistic symptoms can also be correlated to a worse response to antipsychotic medications (93, 94).

Considering all these issues, evaluating the presence of autistic symptoms and their correlates could allow the design and implementation of tailored and targeted treatment and rehabilitation programs, in line with the objective of developing precision medicine also in psychiatry (95).

### Strengths and limitations

A point of strength of the present systematic review is the accuracy of the search, conducted according to the most recent methodological indications (46), and the comprehensive and broad manual search, which also included a search in open databases and citation indices. While some electronic databases (such as Web of Science, CINAHL and CENTRAL) were not searched in a systematic manner, the inclusion of three different databases combined with a thorough Google Scholar search can be considered appropriate for the research question (96). In particular, as the present work focused on the use of the PAUSS scale, checking the citations list of the original validation study allowed a particularly thorough search.

Among the limitations of the present study is the inability to perform meta-analytic analyses: the high level of heterogeneity of the included outcomes and the differences in the use and cut-offs of the scale adopted in the included works did not allow to perform a quantitative synthesis, which could be possible in future studies with a more limited research scope. While the broad research question of the present systematic review led to the inclusion of studies with heterogeneous methods and outcomes, different important aspects and correlates of autistics symptoms were explored, providing valuable insight on the impact of the autistic dimension in people living with schizophrenia.

## Conclusions

The PAUSS scale represents the only validated instrument available to assess the severity autistic symptoms specifically in people living with SSD: since its development, it has been used more and more in the context of clinical research.

The analyzed literature shows that more severe autistic symptoms are associated with worse cognitive performance, especially in the social cognition domain, and with worse psychosocial functioning. However, the presence of autistic symptoms appears to also have a protective role in some instances in subjects with more severe psychotic symptoms.

Future research should focus on studying more thoroughly the correlates on the autistic symptoms, not only in a clinical but also in a neurobiological perspective. It should also focus on longitudinally assessing autistic symptoms and evaluating their stability over time, with the aim of developing tailored care projects and interventions: this could improve the therapeutic and rehabilitation offer for people living with SSD.

## Data availability statement

The original contributions presented in the study are included in the article/Supplementary material, further inquiries can be directed to the corresponding author.

## Author contributions

Conceptualization and methodology: GN, SB, and AV. Data curation: GN, SB, NN EI, and DD'O. Investigation: IC-P, NN, EI, DD'O, and JL. Supervision: GN, SB, GD, and AV. Writing, review and editing: GN, SB, IC-P, and AV. All authors contributed to the article and approved the submitted version.

## Conflict of interest

The authors declare that the research was conducted in the absence of any commercial or financial relationships that could be construed as a potential conflict of interest.

## Publisher's note

All claims expressed in this article are solely those of the authors and do not necessarily represent those of their affiliated organizations, or those of the publisher, the editors and the reviewers. Any product that may be evaluated in this article, or claim that may be made by its manufacturer, is not guaranteed or endorsed by the publisher.

## References

[1] American Psychiatric Association. Diagnostic and Statistical Manual of Mental Disorders, Fifth Edition: DSM-5. Arlington, VA: American Psychiatric Publishing (2013), p. 998. Available online at: http://archive.org/details/diagnosticstatis0005unse (accessed December 19, 2021).

[2] World Health Organization. ICD-11 Guideline GCP Network. (2021). Available online at: https://gcp.network/icd-11-cddr/ (accessed April 29, 2022).

[3] Crespi B Badcock C. Psychosis and autism as diametrical disorders of the social brain. Behav Brain Sci. (2008) 31:241–61. discussion 261–320. 10.1017/S0140525X08004214 18578904

[4] Bleuler E. Dementia praecox or the group of schizophrenias. Madison (1950). 21218204

[5] Evans B. How autism became autism. Hist Hum Sci. (2013) 26:3–31. 10.1177/0952695113484320 24014081 PMC3757918

[6] Kanner L. Autistic disturbances of affective contact. Nerv Child. (1943) 2:217–50. 4880460

[7] Wolff S. The history of autism. Eur Child Adolesc Psychiatry. (2004) 13:201–8. 10.1007/s00787-004-0363-5 15365889

[8] Barlati S Deste G Ariu C Vita A. Autism spectrum disorder and schizophrenia: do they overlap? Int J Emerg Ment Health Hum Resil. (2016) 18:760–3. 10.4172/1522-4821.1000318

[9] De Crescenzo F Postorino V Siracusano M Riccioni A Armando M Curatolo P . Autistic symptoms in schizophrenia spectrum disorders: a systematic review and meta-analysis. Front Psychiatry. (2019) 10:78. 10.3389/fpsyt.2019.00078 30846948 PMC6393379

[10] King BH Lord C. Is schizophrenia on the autism spectrum? Brain Res. (2011) 1380:34–41. 10.1016/j.brainres.2010.11.031 21078305

[11] Rapoport J Chavez A Greenstein D Addington A Gogtay N. Autism spectrum disorders and childhood-onset schizophrenia: clinical and biological contributions to a relation revisited. J Am Acad Child Adolesc Psychiatry. (2009) 48:10–8. 10.1097/CHI.0b013e31818b1c63 19218893 PMC2664646

[12] Barlati S Deste G Gregorelli M Vita A. Autistic traits in a sample of adult patients with schizophrenia: prevalence and correlates. Psychol Med. (2019) 49:140–8. 10.1017/S0033291718000600 29554995

[13] Konstantareas MM Hewitt T. Autistic disorder and schizophrenia: diagnostic overlaps. J Autism Dev Disord. (2001) 31:19–28. 10.1023/A:1005605528309 11439750

[14] Larson FV Wagner AP Jones PB Tantam D Lai M-C Baron-Cohen S . Psychosis in autism: comparison of the features of both conditions in a dually affected cohort. Br J Psychiatry J Ment Sci. (2017) 210:269–75. 10.1192/bjp.bp.116.187682 27979819 PMC5376719

[15] Mouridsen SE Rich B Isager T. Psychiatric disorders in adults diagnosed as children with atypical autism. A case control study. J Neural Transm. (2008) 115:135–138. 10.1007/s00702-007-0798-1 17768593

[16] Barlati S Minelli A Ceraso A Nibbio G Carvalho Silva R Deste G . Social cognition in a research domain criteria perspective: a bridge between schizophrenia and autism spectra disorders. Front Psychiatry. (2020) 11:806. 10.3389/fpsyt.2020.00806 33005149 PMC7485015

[17] Ross CA. Problems with autism, catatonia and schizophrenia in DSM-5. Schizophr Res. (2014) 158:264–5. 10.1016/j.schres.2014.06.017 24999051

[18] Kuo SS Wojtalik JA Mesholam-Gately RI Keshavan MS Eack SM. Transdiagnostic validity of the MATRICS consensus cognitive battery across the autism-schizophrenia spectrum. Psychol Med. (2020) 50:1623–32. 10.1017/S0033291719001582 31298174 PMC9812023

[19] Kuo SS Eack SM. Meta-analysis of cognitive performance in neurodevelopmental disorders during adulthood: comparisons between autism spectrum disorder and schizophrenia on the Wechsler adult intelligence scales. Front Psychiatry. (2020) 11:187. 10.3389/fpsyt.2020.00187 32273855 PMC7114889

[20] Bliksted V Ubukata S Koelkebeck K. Discriminating autism spectrum disorders from schizophrenia by investigation of mental state attribution on an on-line mentalizing task: a review and meta-analysis. Schizophr Res. (2016) 171:16–26. 10.1016/j.schres.2016.01.037 26817402

[21] Chung YS Barch D Strube M. A meta-analysis of mentalizing impairments in adults with schizophrenia and autism spectrum disorder. Schizophr Bull. (2014) 40:602–16. 10.1093/schbul/sbt048 23686020 PMC3984506

[22] Gilleen J Xie F Strelchuk D. 38. Distinct theory of mind deficit profiles in schizophrenia and autism: a meta-analysis of published research Schizophr Bull. (2017) 43:S22. 10.1093/schbul/sbx021.057

[23] Fernandes JM Cajão R Lopes R Jerónimo R Barahona-Corrêa JB. Social cognition in schizophrenia and autism spectrum disorders: a systematic review and meta-analysis of direct comparisons. Front Psychiatry. (2018) 9:504. 10.3389/fpsyt.2018.00504 30459645 PMC6232921

[24] Pinkham AE Morrison KE Penn DL Harvey PD Kelsven S Ludwig K . Comprehensive comparison of social cognitive performance in autism spectrum disorder and schizophrenia. Psychol Med. (2020) 50:2557–65. 10.1017/S0033291719002708 31576783

[25] Eack SM Wojtalik JA Keshavan MS Minshew NJ. Social-cognitive brain function and connectivity during visual perspective-taking in autism and schizophrenia. Schizophr Res. (2017) 183:102–9. 10.1016/j.schres.2017.03.009 28291690 PMC5432384

[26] Park MTM Raznahan A Shaw P Gogtay N Lerch JP Chakravarty MM. Neuroanatomical phenotypes in mental illness: identifying convergent and divergent cortical phenotypes across autism, ADHD and schizophrenia. J Psychiatry Neurosci JPN. (2018) 43:170094. 10.1503/jpn.170094 29688876 PMC5915241

[27] Haigh SM Gupta A Barb SM Glass SAF Minshew NJ Dinstein I . Differential sensory fMRI signatures in autism and schizophrenia: analysis of amplitude and trial-to-trial variability. Schizophr Res. (2016) 175:12–9. 10.1016/j.schres.2016.03.036 27083780 PMC4958557

[28] Dorph-Petersen K-A Lewis DA. Postmortem structural studies of the thalamus in schizophrenia. Schizophr Res. (2017) 180:28–35. 10.1016/j.schres.2016.08.007 27567291 PMC5746188

[29] Nakagawa Y Chiba K. Involvement of neuroinflammation during brain development in social cognitive deficits in autism spectrum disorder and schizophrenia. J Pharmacol Exp Ther. (2016) 358:504–15. 10.1124/jpet.116.234476 27384073

[30] Zuo C Wang D Tao F Wang Y. Changes in the development of subcortical structures in autism spectrum disorder. Neuroreport. (2019) 30:1062–7. 10.1097/WNR.0000000000001300 31464839

[31] Cheung C Yu K Fung G Leung M Wong C Li Q . Autistic disorders and schizophrenia: related or remote? An anatomical likelihood estimation. PloS ONE. (2010) 5:e12233. 10.1371/journal.pone.0012233 20805880 PMC2923607

[32] Schultz RT. Developmental deficits in social perception in autism: the role of the amygdala and fusiform face area. Int J Dev Neurosci. (2005) 23:125–41. 10.1016/j.ijdevneu.2004.12.012 15749240

[33] Toal F Bloemen OJN Deeley Q Tunstall N Daly EM Page L . Psychosis and autism: magnetic resonance imaging study of brain anatomy. Br J Psychiatry J Ment Sci. (2009) 194:418–25. 10.1192/bjp.bp.107.049007 19407271

[34] Schneider M Debbané M Bassett AS Chow EWC Fung WLA van den Bree M. et al. Psychiatric disorders from childhood to adulthood in 22q112 deletion syndrome: results from the international consortium on brain and behavior in 22q112 deletion syndrome. Am J Psychiatry. (2014) 171:627–39. 10.1176/appi.ajp.2013.13070864 24577245 PMC4285461

[35] Tebbenkamp ATN Willsey AJ State MW Sestan N. The developmental transcriptome of the human brain: implications for neurodevelopmental disorders. Curr Opin Neurol. (2014) 27:149–56. 10.1097/WCO.0000000000000069 24565942 PMC4038354

[36] Cardno AG Gottesman II. Twin studies of schizophrenia: from bow-and-arrow concordances to star wars Mx and functional genomics. Am J Med Genet. (2000) 97:12–7. 10.1002/(SICI)1096-8628(200021)97:1<12::AID-AJMG3>3.0.CO;2-U 10813800

[37] de Lacy N King BH. Revisiting the relationship between autism and schizophrenia: toward an integrated neurobiology. Annu Rev Clin Psychol. (2013) 9:555–87. 10.1146/annurev-clinpsy-050212-185627 23537488

[38] Guilmatre A Huguet G Delorme R Bourgeron T. The emerging role of SHANK genes in neuropsychiatric disorders. Dev Neurobiol. (2014) 74:113–22. 10.1002/dneu.22128 24124131

[39] Voineskos AN Lett TAP Lerch JP Tiwari AK Ameis SH Rajji TK . Neurexin-1 and frontal lobe white matter: an overlapping intermediate phenotype for schizophrenia and autism spectrum disorders. PLoS ONE. (2011) 6:e20982. 10.1371/journal.pone.0020982 21687627 PMC3110800

[40] Lord C Rutter M Goode S Heemsbergen J Jordan H Mawhood L . Autism diagnostic observation schedule: a standardized observation of communicative and social behavior. J Autism Dev Disord. (1989) 19:185–212. 10.1007/BF02211841 2745388

[41] Lord C Rutter M Le Couteur A. Autism Diagnostic Interview-Revised: a revised version of a diagnostic interview for caregivers of individuals with possible pervasive developmental disorders. J Autism Dev Disord. (1994) 24:659–85. 10.1007/BF02172145 7814313

[42] Deste G Barlati S Gregorelli M Lisoni J Turrina C Valsecchi P . Looking through autistic features in schizophrenia using the PANSS Autism Severity Score (PAUSS). Psychiatry Res. (2018) 270:764–8. 10.1016/j.psychres.2018.10.074 30551322

[43] Kästner A Begemann M Michel TM Everts S Stepniak B Bach C . Autism beyond diagnostic categories: characterization of autistic phenotypes in schizophrenia. BMC Psychiatry. (2015) 15:115. 10.1186/s12888-015-0494-x 25968177 PMC4436160

[44] Kay SR Fiszbein A Opler LA. The positive and negative syndrome scale (PANSS) for schizophrenia. Schizophr Bull. (1987) 13:261–76. 10.1093/schbul/13.2.261 3616518

[45] Moher D Liberati A Tetzlaff J Altman DG PRISMA Group. Preferred reporting items for systematic reviews and meta-analyses: the PRISMA statement. PLoS Med. (2009) 6:e1000097. 10.1371/journal.pmed.1000097 19621072 PMC2707599

[46] Page MJ McKenzie JE Bossuyt PM Boutron I Hoffmann TC Mulrow CD . The PRISMA 2020 statement: an updated guideline for reporting systematic reviews. BMJ. (2021) 372:n71. 10.1136/bmj.n71 33782057 PMC8005924

[47] Jordan Z Lockwood C Munn Z Aromataris E. The updated Joanna Briggs Institute model of evidence-based healthcare. Int J Evid Based Healthc. (2019) 17:58–71. 10.1097/XEB.0000000000000155 30256247

[48] Munn Z Moola S Lisy K Riitano D Tufanaru C. Methodological guidance for systematic reviews of observational epidemiological studies reporting prevalence and cumulative incidence data. Int J Evid Based Healthc. (2015) 13:147–53. 10.1097/XEB.0000000000000054 26317388

[49] Schünemann H. The GRADE Handbook. Cochrane Collaboration. (2013).

[50] Ribbe K Friedrichs H Begemann M Grube S Papiol S Kästner A . The cross-sectional GRAS sample: a comprehensive phenotypical data collection of schizophrenic patients. BMC Psychiatry. (2010) 10:91. 10.1186/1471-244X-10-91 21067598 PMC3002316

[51] Pinkham AE Harvey PD Penn DL. Social cognition psychometric evaluation: results of the final validation study. Schizophr Bull. (2018) 44:737–48. 10.1093/schbul/sbx117 28981848 PMC6007629

[52] Pinkham AE Penn DL Green MF Harvey PD. Social cognition psychometric evaluation: results of the initial psychometric study. Schizophr Bull. (2016) 42:494–504. 10.1093/schbul/sbv056 25943125 PMC4753585

[53] Galderisi S Rucci P Kirkpatrick B Mucci A Gibertoni D Rocca P . Interplay among psychopathologic variables, personal resources, context-related factors, and real-life functioning in individuals with schizophrenia: a network analysis. JAMA Psychiatry. (2018) 75:396–404. 10.1001/jamapsychiatry.2017.4607 29450447 PMC5875306

[54] Galderisi S Rossi A Rocca P Bertolino A Mucci A Bucci P . The influence of illness-related variables, personal resources and context-related factors on real-life functioning of people with schizophrenia. World Psychiatry. (2014) 13:275–87. 10.1002/wps.20167 25273301 PMC4219069

[55] Vita A Barlati S Deste G Rocca P Rossi A Bertolino A . The influence of autistic symptoms on social and non-social cognition and on real-life functioning in people with schizophrenia: evidence from the Italian Network for Research on Psychoses multicenter study. Eur Psychiatry J Assoc Eur Psychiatr. (2020) 63:e98. 10.1192/j.eurpsy.2020.99 33168115 PMC7737172

[56] Abu-Akel A Wood SJ Upthegrove R Chisholm K Lin A Hansen PC . Psychosocial functioning in the balance between autism and psychosis: evidence from three populations. Mol Psychiatry. (2022) 1−9. 10.1038/s41380-022-01543-5 35422471 PMC9205777

[57] Stefansson H Meyer-Lindenberg A Steinberg S Magnusdottir B Morgen K Arnarsdottir S . CNVs conferring risk of autism or schizophrenia affect cognition in controls. Nature. (2014) 505:361–6. 10.1038/nature12818 24352232

[58] Abu-Akel A Philip RCM Lawrie SM Johnstone EC Stanfield AC. Categorical and dimensional approaches to examining the joint effect of autism and schizotypal personality disorder on sustained attention. Front Psychiatry. (2020) 11:798. 10.3389/fpsyt.2020.00798 32848955 PMC7426517

[59] Barlati S Nibbio G Morena D Cacciani P Corsini P Mosca A . Autistic symptoms in schizophrenia: impact on internalized stigma, well-being, clinical and functional characteristics. Front Psychiatry. (2022) 13:801651. 10.3389/fpsyt.2022.801651 35432047 PMC9005776

[60] Bechi M Agostoni G Buonocore M Bosinelli F Spangaro M Bianchi L . The Influence of premorbid adjustment and autistic traits on social cognitive dysfunction in schizophrenia. J Int Neuropsychol Soc JINS. (2020) 26:276–85. 10.1017/S1355617719000961 31507263

[61] Bechi M Agostoni G Buonocore M Gritti D Mascia M Spangaro M . The association of autistic traits with Theory of Mind and its training efficacy in patients with schizophrenia. Schizophr Res Cogn. (2020) 19:100164. 10.1016/j.scog.2019.100164 31832344 PMC6890977

[62] Bechi M Abu-Akel A Agostoni G Bosia M Cocchi F Spangaro M . Functional benefits of co-occurring autistic symptoms in schizophrenia is delimited by symptom severity. J Psychiatr Res. (2021) 137:48–54. 10.1016/j.jpsychires.2021.02.044 33652326

[63] Bechi M Abu-Akel A Agostoni G Buonocore M Bosia M Martini F Cavallaro R. Theory of mind and stereotypic behavior promote daily functioning in patients with schizophrenia. Aust N Z J Psychiatry. (2020) 56:818–27. 10.1177/00048674211038513 34376088

[64] Deste G Vita A Penn DL Pinkham AE Nibbio G Harvey PD. Autistic symptoms predict social cognitive performance in patients with schizophrenia. Schizophr Res. (2020) 215:113–9. 10.1016/j.schres.2019.11.008 31780344 PMC7035981

[65] Deste G Vita A Nibbio G Penn DL Pinkham AE Harvey PD. Autistic symptoms and social cognition predict real-world outcomes in patients with schizophrenia. Front Psychiatry. (2020) 11:524. 10.3389/fpsyt.2020.00524 32581892 PMC7294984

[66] Deste G Vita A Nibbio G Barlati S Penn DL Pinkham AE . Autistic symptoms in people with schizophrenia: neurocognitive, socio-cognitive, clinical and real-world functional characteristics of individuals without autistic features. Schizophr Res. (2021) 236:12–8. 10.1016/j.schres.2021.07.037 34364032

[67] Ehrenreich H Mitjans M Van der Auwera S Centeno TP Begemann M Grabe HJ . a new strategy to extract mental disease-relevant combinations of GWAS hits from individuals. Mol Psychiatry. (2018) 23:476–86. 10.1038/mp.2016.208 27922606 PMC5794905

[68] Harvey PD Deckler E Jones MT Jarskog LF Penn DL Pinkham AE. Autism symptoms, depression, and active social avoidance in schizophrenia: association with self-reports and informant assessments of everyday functioning. J Psychiatr Res. (2019) 115:36–42. 10.1016/j.jpsychires.2019.05.010 31102902 PMC6556410

[69] Mitjans M Begemann M Ju A Dere E Wüstefeld L Hofer S . Sexual dimorphism of AMBRA1-related autistic features in human and mouse. Transl Psychiatry. (2017) 7:e1247. 10.1038/tp.2017.213 28994820 PMC5682605

[70] Oliveira B Mitjans M Nitsche MA Kuo M-F Ehrenreich H. Excitation-inhibition dysbalance as predictor of autistic phenotypes. J Psychiatr Res. (2018) 104:96–9. 10.1016/j.jpsychires.2018.06.004 30015265

[71] Palumbo D Stanghellini G Mucci A Ballerini M Giordano GM Lysaker PH . Autism rating scale: a new tool for characterizing the schizophrenia phenotype. Front Psychiatry. (2021) 12:622359. 10.3389/fpsyt.2021.622359 33574776 PMC7870791

[72] Parellada M Pina-Camacho L Moreno C Aleman Y Krebs M-O Desco M . Insular pathology in young people with high-functioning autism and first-episode psychosis. Psychol Med. (2017) 47:2472–82. 10.1017/S0033291717000988 28436341

[73] Pina-Camacho L Boada L Díaz-Caneja CM García-Alcón A Burdeus M Serrano-Drozdowskyj E . The Positive and Negative Syndrome Scale for Schizophrenia Autism Severity Scale (PAUSS) in young people with autism and schizophrenia. Rev Psiquiatr Salud Ment. (2020) 13:118–30. 10.1016/j.rpsm.2020.05.006 32703733

[74] Stepniak B Kästner A Poggi G Mitjans M Begemann M Hartmann A . Accumulated common variants in the broader fragile X gene family modulate autistic phenotypes. EMBO Mol Med. (2015) 7:1565–79. 10.15252/emmm.201505696 26612855 PMC4693501

[75] Vaskinn A Abu-Akel A. The interactive effect of autism and psychosis severity on theory of mind and functioning in schizophrenia. Neuropsychology. (2019) 33:195–202. 10.1037/neu0000499 30346197

[76] Barlati S Morena D Nibbio G Cacciani P Corsini P Mosca A . Internalized stigma among people with schizophrenia: relationship with socio-demographic, clinical and medication-related features. Schizophr Res. (2021) 243:364–71. 10.1016/j.schres.2021.06.007 34183209

[77] Catillon M. Trends and predictors of biomedical research quality, 1990-2015: a meta-research study. BMJ Open. (2019) 9:e030342. 10.1136/bmjopen-2019-030342 31481564 PMC6731820

[78] Vinkers CH Lamberink HJ Tijdink JK Heus P Bouter L Glasziou P . The methodological quality of 176,620 randomized controlled trials published between 1966 and 2018 reveals a positive trend but also an urgent need for improvement. PLoS Biol. (2021) 19:e3001162. 10.1371/journal.pbio.3001162 33872298 PMC8084332

[79] Hyatt CJ Wexler BE Pittman B Nicholson A Pearlson GD Corbera S . Atypical dynamic functional network connectivity state engagement during social-emotional processing in schizophrenia and autism. Cereb Cortex N Y N. (2021) bhab423. 10.1093/cercor/bhab423 34875687 PMC9376868

[80] Ziermans TB Schirmbeck F Oosterwijk F Geurts HM de Haan L Genetic Risk and Outcome of Psychosis (GROUP) Investigators. Autistic traits in psychotic disorders: prevalence, familial risk, and impact on social functioning. Psychol Med. (2021) 51:1704–13. 10.1017/S0033291720000458 32151297 PMC8327624

[81] Dell'Osso L Carpita B Cremone IM Gesi C D'Ermo A De Iorio G . Autism spectrum in patients with schizophrenia: correlations with real-life functioning, resilience, and coping styles. CNS Spectr. (2021) 1–11. 10.1017/S1092852921000353 33843551

[82] Isvoranu A-M Ziermans T Schirmbeck F Borsboom D Geurts HM de Haan L . Autistic symptoms and social functioning in psychosis: a network approach. Schizophr Bull. (2022) 48:273–82. 10.1093/schbul/sbab084 34313767 PMC8781349

[83] Galderisi S Mucci A Dollfus S Nordentoft M Falkai P Kaiser S . EPA guidance on assessment of negative symptoms in schizophrenia. Eur Psychiatry. (2021) 64:e23. 10.1192/j.eurpsy.2021.11 33597064 PMC8080207

[84] Rossi A Galderisi S Rocca P Bertolino A Rucci P Gibertoni D . Personal resources and depression in schizophrenia: the role of self-esteem, resilience and internalized stigma. Psychiatry Res. (2017) 256:359–64. 10.1016/j.psychres.2017.06.079 28686933

[85] Sampogna G Henderson C Thornicroft G Evans-Lacko S Bakolis I Robinson E . Are social networks useful to challenge stigma attached to mental disorders? Findings from the time to change social marketing campaign 2009–2014. Eur Psychiatry. (2017) 41:S89. 10.1016/j.eurpsy.2017.01.279 27997875

[86] Yanos PT DeLuca JS Roe D Lysaker PH. The impact of illness identity on recovery from severe mental illness: a review of the evidence. Psychiatry Res. (2020) 288:112950. 10.1016/j.psychres.2020.112950 32361335

[87] Komatsu H Ono T Onoguchi G Tomita H Kakuto Y. Mediating effects of self-stigma and depression on the association between autistic symptoms and recovery in patients with schizophrenia-spectrum disorders: a cross-sectional study. BMC Psychiatry. (2021) 21:464. 10.1186/s12888-021-03472-z 34556056 PMC8461904

[88] Komatsu H Ono T Maita Y Ishida Y Kikuchi T Maki T . Association between autistic symptoms and self-stigma in patients with schizophrenia spectrum disorders. Neuropsychiatr Dis Treat. (2020) 16:2553–61. 10.2147/NDT.S280485 33154642 PMC7605940

[89] Vita A Barlati S Ceraso A Deste G Nibbio G Wykes T. Acceptability of cognitive remediation for schizophrenia: a systematic review and meta-analysis of randomized controlled trials. Psychol Med. (2022) 1–11. 10.1017/S0033291722000319 35257646 PMC10277755

[90] Vita A Barlati S Ceraso A Nibbio G Ariu C Deste G . Effectiveness, core elements, and moderators of response of cognitive remediation for schizophrenia: a systematic review and meta-analysis of randomized clinical trials. JAMA Psychiatry. (2021) 78:848–58. 10.1001/jamapsychiatry.2021.0620 33877289 PMC8058696

[91] Nibbio G Barlati S Cacciani P Corsini P Mosca A Ceraso A . Evidence-based integrated intervention in patients with schizophrenia: a pilot study of feasibility and effectiveness in a real-world rehabilitation setting. Int J Environ Res Public Health. (2020) 17:E3352. 10.3390/ijerph17103352 32408561 PMC7277196

[92] Wykes T Huddy V Cellard C McGurk SR Czobor P. A Meta-analysis of cognitive remediation for schizophrenia: methodology and effect sizes. Am J Psychiatry. (2011) 168:472–85. 10.1176/appi.ajp.2010.10060855 21406461

[93] Downs JM Lechler S Dean H Sears N Patel R Shetty H . The association between comorbid autism spectrum disorders and antipsychotic treatment failure in early-onset psychosis: a historical cohort study using electronic health records. J Clin Psychiatry. (2017) 78:e1233–41. 10.4088/JCP.16m11422 29125721 PMC6037287

[94] Nakata Y Kanahara N Kimura A Niitsu T Komatsu H Oda Y . Autistic traits and cognitive profiles of treatment-resistant schizophrenia. Schizophr Res Cogn. (2020) 22:100186. 10.1016/j.scog.2020.100186 32760657 PMC7390750

[95] Maj M van Os J De Hert M Gaebel W Galderisi S Green MF . The clinical characterization of the patient with primary psychosis aimed at personalization of management. World Psychiatry. (2021) 20:4–33. 10.1002/wps.20809 33432763 PMC7801854

[96] Bramer WM Rethlefsen ML Kleijnen J Franco OH. Optimal database combinations for literature searches in systematic reviews: a prospective exploratory study. Syst Rev. (2017) 6:245. 10.1186/s13643-017-0644-y 29208034 PMC5718002

