# Supplementary material for: Assessment and correlates of autistic symptoms in Schizophrenia Spectrum Disorders measured with the PANSS Autism Severity Score: A systematic review
Source: Front Psychiatry. 2022 Aug 30;13:934005. doi: 10.3389/fpsyt.2022.934005 (PMC9468543; doi:10.3389/fpsyt.2022.934005)
Supplement: Supplementary file 1 [file Data_Sheet_1.docx]

**Appendix 1. Search string for specific databases**

**PubMed/MEDLINE:**

("schizophrenia"[MeSH Terms] OR "schizophrenia"[All Fields] OR "schizophrenias"[All Fields] OR "schizophrenia’s"[All Fields] OR "SSD"[All Fields] OR "psycho*"[All Fields]) AND ("PAUSS"[All Fields] OR "PANSS autism severity score"[All Fields])

**Scopus**

ALL ( ( schizophrenia OR "SSD" OR "psycho*" ) AND ( "PAUSS" OR "PANSS autism severity score" ) )

**PsycINFO**

TI ((schizophrenia OR “SSD” OR “psycho*”) AND (“PAUSS” OR “PANSS autism severity score”)) OR AB ((schizophrenia OR “SSD” OR “psycho*”) AND (“PAUSS” OR “PANSS autism severity score”)) OR TX ((schizophrenia OR “SSD” OR “psycho*”) AND (“PAUSS” OR “PANSS autism severity score”))

**Appendix 2. Methodological quality assessment according to Joanna Briggs Institute (JBI) checklist**

| **Publication**  **(Cross-sectional)** | **JBI Item 1** | **JBI Item 2** | **JBI Item 3** | **JBI Item 4** | **JBI Item 5** | **JBI Item 6** | **JBI Item 7** | **JBI Item 8** |
| --- | --- | --- | --- | --- | --- | --- | --- | --- |
| Abu-Akel et al., 2020 | Yes | Yes | Yes | Yes | Yes | Yes | Yes | Yes |
| Abu-Akel et al., 2022 | Yes | Yes | Yes | Yes | Yes | Yes | Yes | Yes |
| Barlati et al., 2022 | Yes | Yes | Yes | Yes | Yes | Yes | Yes | Yes |
| Bechi et al., 2019 | Yes | Yes | Yes | Yes | Yes | No | Yes | Yes |
| Bechi et al., 2020a | Yes | Yes | Yes | Yes | Yes | Yes | Yes | Yes |
| Bechi et al., 2021a | Yes | Yes | Yes | Yes | Yes | Yes | Yes | Yes |
| Bechi et al., 2021b | Yes | Yes | Yes | Yes | Yes | Yes | Yes | Yes |
| Deste et. al., 2018 | Yes | Yes | Yes | Yes | No | No | Yes | Yes |
| Deste et al., 2020a | Yes | Yes | Yes | Yes | Yes | Yes | Yes | Yes |
| Deste et al., 2020 b | Yes | Yes | Yes | Yes | Yes | Yes | Yes | Yes |
| Deste et al., 2021 | Yes | Yes | Yes | Yes | Yes | Yes | Yes | Yes |
| Ehrenreich et al., 2018 | Yes | Yes | Yes | Yes | No | No | Yes | Yes |
| Harvey et al., 2019 | Yes | Yes | Yes | Yes | No | No | Yes | Yes |
| Kastner et al., 2015 | Yes | Yes | Yes | Yes | No | No | Yes | Yes |
| Mitjans et al., 2017 | Yes | Yes | Yes | Yes | No | No | Yes | Yes |
| Oliveira et al., 2018 | Yes | Yes | Yes | Yes | Yes | Yes | Yes | Yes |
| Palumbo et al., 2021 | Yes | Yes | Yes | Yes | Yes | No | Yes | Yes |
| Parellada et al., 2017 | Yes | Yes | Yes | Yes | Yes | No | Yes | Yes |
| Pina – Camacho et al., 2020 | Yes | Yes | Yes | Yes | Yes | Yes | Yes | Yes |
| Stepniak et al., 2015 | Yes | Yes | Yes | Yes | Yes | Yes | Yes | Yes |
| Vaskinn e Abu-Akel, 2019 | Yes | Yes | Yes | Yes | Yes | Yes | Yes | Yes |
| Vita et al., 2020 | Yes | Yes | Yes | Yes | Yes | Yes | Yes | Yes |

| **Publication**  **(Non-controlled trials)** | **JBI Item 1** | **JBI Item 2** | **JBI Item 3** | **JBI Item 4** | **JBI Item 5** | **JBI Item 6** | **JBI Item 7** | **JBI Item 8** | **JBI Item 9** |
| --- | --- | --- | --- | --- | --- | --- | --- | --- | --- |
| Bechi et al., 2020b | Yes | Yes | Yes | No | Yes | Yes | Yes | Yes | Yes |
| Oliveira et al., 2018 | Yes | Yes | Yes | No | Not applicable | Not applicable | Yes | Yes | Yes |
